# Topological regularization of networks in temporal lobe epilepsy: a structural MRI study

**DOI:** 10.3389/fnins.2024.1423389

**Published:** 2024-07-04

**Authors:** Yini Chen, Lu Sun, Shiyao Wang, Beiyan Guan, Jingyu Pan, Yiwei Qi, Yufei Li, Nan Yang, Hongsen Lin, Ying Wang, Bo Sun

**Affiliations:** ^1^Department of Radiology, The First Affiliated Hospital of Dalian Medical University, Dalian, China; ^2^Department of Neurology, The First Affiliated Hospital, Dalian Medical University, Dalian, China

**Keywords:** temporal lobe epilepsy, connectome, graph theory, gray matter volume, structural covariance network

## Abstract

**Objective:**

Patients with temporal lobe epilepsy (TLE) often exhibit neurocognitive disorders; however, we still know very little about the pathogenesis of cognitive impairment in patients with TLE. Therefore, our aim is to detect changes in the structural connectivity networks (SCN) of patients with TLE.

**Methods:**

Thirty-five patients with TLE were compared with 47 normal controls (NC) matched according to age, gender, handedness, and education level. All subjects underwent thin-slice T1WI scanning of the brain using a 3.0 T MRI. Then, a large-scale structural covariance network was constructed based on the gray matter volume extracted from the structural MRI. Graph theory was then used to determine the topological changes in the structural covariance network of TLE patients.

**Results:**

Although small-world networks were retained, the structural covariance network of TLE patients exhibited topological irregularities in regular architecture as evidenced by an increase in the small world properties (*p* < 0.001), normalized clustering coefficient (*p* < 0.001), and a decrease in the transfer coefficient (*p* < 0.001) compared with the NC group. Locally, TLE patients showed a decrease in nodal betweenness and degree in the left lingual gyrus, right middle occipital gyrus and right thalamus compared with the NC group (*p* < 0.05, uncorrected). The degree of structural networks in both TLE (Temporal Lobe Epilepsy) and control groups was distributed exponentially in truncated power law. In addition, the stability of random faults in the structural covariance network of TLE patients was stronger (*p* = 0.01), but its fault tolerance was lower (*p* = 0.03).

**Conclusion:**

The objective of this study is to investigate the potential neurobiological mechanisms associated with temporal lobe epilepsy through graph theoretical analysis, and to examine the topological characteristics and robustness of gray matter structural networks at the network level.

## Introduction

Epilepsy (EP) was the second most common neurological disorder, characterized by frequent and unexplained seizures (Téllez-Zenteno and Hernández-Ronquillo, 2012; Singh and Trevick, 2016). The essence of an epileptic seizure was the transient abnormal discharge of brain neurons, leading to specific clinical signs or symptoms. Temporal lobe epilepsy (TLE) originates from the temporal lobe region, accounting for approximately 40% of all seizures, making it one of the most common and severe types of epilepsy. Its clinical feature was the progressive development of spontaneous recurrent seizures; thus, it was intractable, recurrent, and drug-resistant (Téllez-Zenteno and Hernández-Ronquillo, 2012). Patients with TLE often exhibited cognitive impairment, especially in memory and executive functions (Mameniškienė et al., 2016). Cognitive impairment (CI) was reported in 30–40% of patients with epilepsy, which may lead to a deterioration in quality of life and eventual disability (Kanner et al., 2020). The degree of cognitive impairment varied from individual to individual and may worsen over time. Increasingly, evidence suggested that TLE was an abnormal network disease of epilepsy, not just a pathogen; epilepsy could be conceptualized as a network disorder (González Otárula and Schuele, 2020; Tabibian et al., 2023). The cognitive state of patients with TLE changed with alterations in brain structure, but the neuroimaging mechanism underlying neurocognitive impairment in these patients remains unclear.

Existing studies had established grey matter disparities in patients with TLE (Alvim et al., 2016). Certain studies showed anomalies in grey matter regions such as the hippocampus (Marsh et al., 2005), amygdala (Sone et al., 2016), and putamen (Kenchaiah et al., 2020) in patients with TLE. The brain regions concerned might not concur across various studies, arguably due to methodological variances, discrepancies in sample selection, and data analysis, leading to different outcomes. Some studies, for instance, found the swelling and enlargement of the amygdala in patients with TLE (Sporns et al., 2005; Sone et al., 2017; Peedicail et al., 2020), while others indicate amygdala atrophy (Armstrong, 1993; Guerreiro et al., 2005; Goldberg et al., 2014; Hakyemez et al., 2016). These differences could be attributed to the pathophysiological underpinnings of TLE. While traditional studies are capable of detecting volume changes in multiple grey matter regions in TLE patients, they fell short of elucidating the structural or functional connections among these regions, failing to provide these changes at a comprehensive brain network level.

In an attempt to reach a thorough comprehension of the complex network of the human brain, Sporns and colleagues (Sporns et al., 2005) proposed the concept of the human connectome. The concept delved into the intricacies of the organizational patterns within the brain in detail. Consequently, the perception of the brain had evolved from a collection of discrete anatomical structures or chemicals to a complex network of interconnected neurons. The paradigm shift in this concept has enabled a novel avenue for in-depth investigation of neural activity in the brain and the pathogenesis of a myriad of neuropsychiatric disorders. The aim of human connectomics was to depict the human brain network map in a comprehensive and detailed manner. It covered the spectrum from a macroscopic level (brain region) to a microscopic level (single neuron), additionally exploring the rules of network connectivity (Sporns et al., 2005; Liang et al., 2021). Through human connectomics, it was possible to interpret brain network connections via three spatial dimensions: microscale, mesoscale, and macroscale, which denote neurons, neuron clusters and brain regions, respectively. In the context of identifying and characterising human brain networks, graph theory emerges as a standard method. It facilitated an intuitive and effective modelling of the intricate structure and functionality of the brain (Marsh et al., 2005; Alvim et al., 2016; González Otárula and Schuele, 2020). By employing this method, researchers could delve into the topological characteristics of human brain networks, such as node connectivity, graph clustering coefficients and path length. This in-depth understanding achieved through graph theory aids in unveiling the organizational principles governing human brain networks, pinpointing crucial neurons or brain regions, and illustrating the interaction dynamics amidst different networks (Marsh et al., 2005; Alvim et al., 2016).

The structural T1-weighted imaging, compared to functional magnetic resonance imaging (fMRI) and diffusion magnetic resonance imaging (dMRI), was an often-seen sequence in a variety of clinical imaging protocols. It offered a faster image acquisition rate and experiences minimal distractions. Such images typically remain unaffected by the distortion and signal attenuation artifacts that are regularly observed in frontoorbital and temporal base regions during functional echo plane imaging and diffusion magnetic resonance imaging (Bernhardt et al., 2013; Byeon et al., 2015; Zaki et al., 2018). Employing nodes to signified different regions of the cerebral cortex, the structural covariance network (SCN) based on graph theory represents the correlation of morphological measurements (e.g., cortical thickness) amongst these areas to construct a connected structure of brain networks. This analysis was defined not by the resolution of imaging voxels, rather by the sampling density of points on the cortex surface (Luo et al., 2019, 2022). As such, it could outline the correlation between structures across the whole brain region. SCN analysis had been put to use in the research of various central nervous system diseases (Han et al., 2021; Saviola et al., 2021; Faridi et al., 2022; Eussen et al., 2023; Shi et al., 2023) and was viewed as a potentially effective tool for investigating brain network alterations in epilepsy (Ai et al., 2023).Although previous studies have conducted graph theory research on patients with temporal lobe epilepsy, these studies have all used functional imaging such as resting-state imaging and DTI to characterize changes in the brain networks of patients with temporal lobe epilepsy (Bernhardt et al., 2013; Lin et al., 2020). Despite these studies effectively revealing abnormalities in the brain networks of patients with temporal lobe epilepsy, these functional sequences are prone to artifact interference and long scanning times. Therefore, this study plans to use structural magnetic resonance imaging for graph theory analysis, attempting to explore potential network abnormalities in patients with temporal lobe epilepsy from the perspective of connectomics. In efforts to delve into the topological characteristics of cortical thickness in patients with TLE, a group of patients clinically diagnosed with TLE were examined alongside a normal control group (NC) matched for age, handedness, sex, and education level using SCN.

To this end, we used graph theory to analyze the changes in the topological properties of structural covariance networks in TLE patients. We further explored the neuroimaging mechanism in TLE patients with cognitive impairment reveal new imaging evidence for the TLE-based cognitive impairment.

## Patients and methods

### Participants

A senior neurologist with 16 years of experience used the 2017 International League Against Epilepsy (ILAE) focal epilepsy diagnostic criteria to determine the diagnosis of all patients (Fisher et al., 2005). This process included a comprehensive evaluation of seizure history, physical examination, EEG examination, imaging examination, lab tests, and other auxiliary exams. A separate senior neurologist, with 17 years of experience, independently reviewed the MRI images of all participants to ascertain the absence of epileptogenic lesions such as malformations, tumors, and reactive gliosis. Any participants with a history of previous brain surgery, chronic medical conditions aside from epilepsy, MRI contraindications, substance abuse, or mental health disorders were excluded from the study. Professional psychoanalysts conducted neuropsychological tests on all subjects. They used the Mini Mental State Examination form (MMSE) and the Montreal Cognitive Assessment (MoCA) to measure cognitive function. The Hamilton Anxiety Scale (HAMA) and Hamilton Depression Scale (HAMD) were utilised for psychological evaluations. Ultimately, 35 patients with temporal lobe epilepsy were included in the study.

In this study, we enlisted 47 healthy volunteers as control subjects, each of whom was matched in age, sex, handedness, and level of education to the patients. The entire research scheme has received approval from the Ethics Committee of the First Affiliated Hospital of Dalian Medical University. After obtaining the approval of the ethics committee, the patient’s written informed consent was waived.

### Imaging data acquisition

In this study, all images were retrospectively collected from uMR Omega 3.0 T MR (uMR Jupiter; United Imaging Healthcare) and Philips Ingenia CX 3.0 T (Philips Healthcare, Best, the Netherlands) scanners. These devices, fitted with 32-channel head coils, enabled us to acquire brain MRI images. All of our subjects were scanned in a supine position, and they were instructed to maintain stillness throughout the scanning procedure. The 3D T1WI sequence parameters were as follows: For the uMR Omega, repetition time = 9.0 ms, echo time = 3.6 ms, voxel size = 0.5 × 0.5 × 0.5 mm^3^, slice thickness = 0.5 mm, matrix size = 512 × 360, and number of slices = 440. For the Philips Ingenia, repetition time = 8.4 ms, echo time = 3.8 ms, voxel size = 1 × 1 × 1 mm^3^, matrix size = 200 × 200, and number of slices = 220. The structural images derived from these scans served the essential purpose of identifying and ruling out possible intracranial structural abnormalities such as severe white matter lesions, cerebrovascular conditions, brain atrophy, cerebral infarction, ectopic gray matter, and cerebral hemorrhages. Details of participants collected by different devices are shown in Supplementary Table S1.

### Image preprocessing

First, a senior imaging diagnosticians examined the T1WI images of each subject and marked those with poor image quality (false artifacts and incomplete images) and parenchymal lesions for exclusion. Then the T1WI images were preprocessed using the VBM toolbox^1^ in the SPM8 software package implemented in MATLAB 2013b (Mathworks, Natick, MA, United States).The specific steps were as follows: (1) Data format conversion: converting a DICOM format image into a NIFTI format image; (2) segmentation: All T1WI images were segmented into gray matter (GM), white matter (WM) and cerebrospinal fluid; (3) normalization: the GM image was registered to the Montreal Neurological Institute (MNI template) based on the DARTEL algorithm; (4) modulation: the deviation generated in the process of standardization was corrected, which produced the final modulated GM image.

### Construction of the structural covariance network

According to Cao et al., each brain was divided into 90 cortical and subcortical regions of interest (ROI), excluding the cerebellum, using the automatic anatomical labeling (AAL) template (Rolls et al., 2020). Firstly, the average volume of each Region of Interest (ROI) was obtained from the Grey Matter (GM) map created by Voxel-based Morphometry (VBM). Following this, a linear regression analysis was facilitated for each ROI to offset the influences of factors such as age, gender, and total intracranial volume (TIV). The residuals resulting from this regression, otherwise known as the corrected GM volume, was then utilized in the construction of a structural covariance network. To accomplish this, we employed the Pearson correlation coefficient to assess the relationships between individual corrected GM volumes, subsequently generating a correlation matrix [Rij], where I and J range from 1 to N (*N* = 90) for each group. This correlation matrix was then refined by resetting diagonal elements and negative correlations to zero, exclusively preserving the positive correlation values. By defining a specific threshold within the correlation matrix R, we obtained a binary adjacency matrix [Aij]. This was done by setting Aij = 1 if [Rij] was greater than the threshold, otherwise, Aij = 0. This resulted in adjacency matrix A, which symbolizes a binary, undirected graph G, with N representing the number of nodes and E symbolizing the count of edges. In this context, the nodes mirror specific regions of the brain, and the edges correspond to undirected neural connections. Non-zero elements in A further represent these connections.

### Structural covariance network analysis

The Graph Analysis Toolbox (GAT) (Lambiotte et al., 2012), based on graph theory, was used to calculate and compare the topological properties of the structural covariance network between the TLE and NC groups. In this study, we used GAT to construct structural covariant networks based on the volume of non-gray matter, and further calculate the topological properties of these networks. It was noteworthy that we included the age, gender, and TIV of each participant as covariates in the analysis to obtain more accurate results. This helped to understand the connection patterns between different regions of the brain and how they work together. The calculation indicators were detailed in the Supplementary material.

### Selection of network density range

The conundrum here is that applying an absolute threshold to the correlation matrix can result in uneven numbers of nodes and edges within the two network groups, thereby complicating succeeding group comparisons. To circumvent this predicament, we employed a uniform network density (D) range for group comparisons. Here, D is defined as the ratio of the actual number of edges to the theoretical maximum edge count. For the purpose of this study, we decided on a network density range of 0.22 ≤ D ≤ 0.5. Concretely, the lower bound of this range is representative of the minimum density wherein both network groups lack isolated nodes (Dmin = 0.22). Conversely, the upper bound signifies the maximum density limit (Dmax = 0.5), beyond which the network begins to emulate random network characteristics.

### Global network parameters

At the overarching global level, our study considered several globally acknowledged attribute analysis indicators. These encompass (Vecchio et al., 2014; Xu et al., 2019):

1. The Normalized Clustering Coefficient (*γ*). It serves to gauge the network’s ‘separation’ function;
2. The Normalized Characteristic Path Length (λ). This measurement plays a role in determining the ‘integration’ function of the network;
3. The Small World Properties (σ). This attribute considers the balance between both ‘separation’ and ‘integration’ functions within the network;
4. The Clustering Coefficient (Cp). This metric assesses the extent of the network’s collectivization;
5. The Characteristic Path Length (Lp). This provides a measure of the resources required for information transmission within the system;
6. The Transfer Coefficient (T). Defined as the variability of the clustering index computed at a global level;
7. The Global Efficiency (Eg). This metric evaluates the network’s overall data conveyance capabilities, and finally;
8. The Local Efficiency (Eloc). Advocating for a measure of the network’s local information transmission efficiency.

Please refer to Supplementary Table S2 for a comprehensive list of definitions and significances attached to the globally-relevant attribute parameters employed in the study of the brain network.

### Regional network parameter

Nodal degree computed the sum of weights of edges connected to the node, which was a simple measurement of connectivity of a node with the rest of the nodes in the network (Park et al., 2017; Wang et al., 2022).Examining the network at the node level, we have opted for the following attributes for analysis:

1. The Betweenness Centrality (Bc) of a node, a metric that measures the influence of a specific node on the flow of information between other nodes in the network;
2. The Degree Centrality (Dc) of a node, a measure that quantifies the capability of a given node to communicate information within the functional network.

### Degree distribution

Degree distribution showed the degree patterns of all the nodes in the network, indicating the resilience of specific network from random failure and targeted attack (Lambiotte et al., 2012). If a network retained a high degree distribution, it might be vulnerable to network impairment (Albert et al., 2000; He et al., 2008). Previous studies had revealed that if a structural covariance network meets a truncated power-law distribution (He et al., 2008; Lambiotte et al., 2012), it might contain a number of areas with mean nodal degree, and also a few of areas with higher nodal degree. The formulation for deducing the distribution was as P(d) ~ [d (e − 1) * exp. (−d/dc)], where P(d) was the probability of the network area degree (d), dc was the cutoff degree, showing the boundary for the probability of the nodes with high degree to be exponentially decayed, e was the exponent representing the scaling scheme (Lambiotte et al., 2012). To minimize the influence of noise on smaller data sets, the cumulative degree distribution was adopted (Xu et al., 2019).

### Network fault tolerance and anti-aggression analysis

In the context of human brain networks, ‘fault tolerance’ and ‘anti-aggression’ alluded to the network’s resilience in the face of random faults and targeted attacks. ‘Targeted attack’ was defined here as the deliberate deletion of nodes in descending order of nodal degree, while a ‘random fault’ implies the random deletion of nodes in the network (Sporns et al., 2005; Bullmore and Sporns, 2009). This study utilized a model that either randomly or specifically deleted nodes to evaluate network fault tolerance and anti-aggression capabilities. In the analysis of fault tolerance, we measured the variations in the network’s largest remaining component after certain nodes were excluded. To ensure the stability of the results, each analysis was repeated 1,000 times. Regarding anti-aggression analysis, we gauged the alteration in the largest remaining network component after eliminating nodes in decreasing order of degree.

### Statistical analysis

In this study, we utilized R language,^2^ a free software environment for statistical computing and graphics, to analyze the demographic and clinical data across the two groups. Depending on the nature of the data, we applied either the Independent sample t-test or nonparametric test for intergroup comparison of measurement data. Data conforming to a normal distribution were expressed as mean ± standard deviation, whereas, for data not conforming to a normal distribution, we used the median and interquartile range to characterize them. In the non-parametric permutation approach, the study reassigned the group labels to create a correlation matrix for randomly formed groups. This correlation matrix was then thresholded across various network densities to derive a binary correlation matrix. Subsequently, the network properties of all binary correlation matrices were computed, and the differences between random groups at each network density were assessed. The divergence between the original and randomly assembled groups constituted a permutation map. Age, sex and TIV were included as covariates in the calculations to eliminate potential confounds. Based on the actual difference value, the *p*-value was ascertained at the pertinent position on the permutation map. Furthermore, Global and local network parameters were calculated using Graph Attention Networks (GAT), and the Area Under the Curve (AUC) value of the global network parameters was appraised. To address multiple comparisons, corrections were applied utilizing the False Discovery Rate (FDR) method. A statistical significance level of *p* < 0.05 was set for inter-group differences.

## Results

### Demographic data and clinical data

Table 1 encapsulated the clinical data pertaining to the TLE patients and NC group. Pertinently, the observed variables including sex, age, and education level exhibited no significant discrepancy between the two groups. However, clear differential patterns were noted in the scores of MMSE, MoCA, HAMA, and HAMD across the TLE and NC groups — all with a significant level of difference (*p* < 0.001).

**Table 1:** Clinical data of the TLE and NC groups.

Characteristics	NC (*n* = 47)	TLE (*n* = 35)	Statistic	*p*
Age at examination/year	58.00 (34.00, 65.50)	47.00 (35.50, 52.50)	*Z* = −1.73	0.084
Gender, *n* (%)			*χ*^2^ = 1.55	0.213
Male	19 (40.43)	19 (54.29)		
Female	28 (59.57)	16 (45.71)		
MMSE	28.00 (28.00, 29.00)	27.00 (24.00, 28.00)	*Z* = −4.00	<0.001^***^
MoCA	27.00 (25.00, 27.00)	22.00 (18.00, 26.00)	*Z* = −4.65	<0.001^***^
HAMA	5.00 (4.00, 6.00)	8.00 (6.00, 12.00)	*Z* = −3.74	<0.001^***^
HAMD	3.00 (2.00, 6.00)	8.00 (5.00, 10.50)	*Z* = −4.28	<0.001^***^
Education/year	12.00 (9.00, 16.00)	12.00 (9.00, 15.00)	*Z* = −0.28	0.780
Disease duration/month	–	36.00(12.00,168.00)	–	–

*Z*, Mann–Whitney test; *χ*^2^: Chi-square test, MMSE, Minimum Mental State Examination; MoCA, Hamilton Anxiety Scale; HAMD, Hamilton Depression Scale; ^***^p < 0.001.

### Comparison of global network metrics

**Figure 1:**
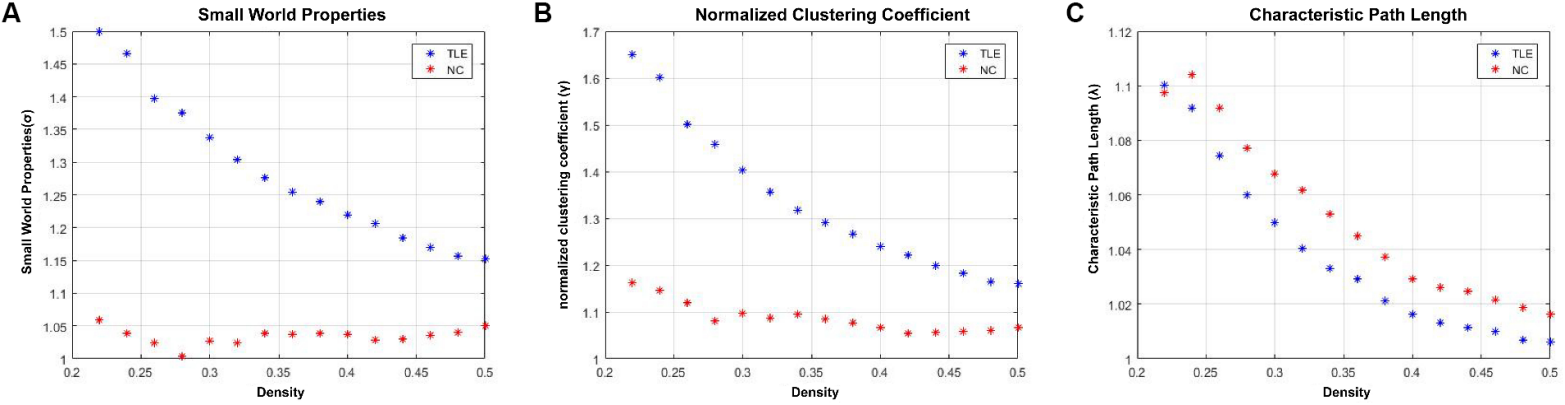
The small world parameters of the structural covariance network of TLE group and NC group. In the network density range of 0.22–0.50, the normalized clustering coefficient (*γ*) **(B)** of the TLE and NC groups was significantly greater than 1, the normalized characteristic path length (λ) **(C)** was approximately equal to 1, and the small world properties (σ) **(A)** was significantly greater than 1. These values indicate that the structure of the covariant network of the TLE and NC group presents typical “small world” network attributes.

In the network density of D = 0.22 ~ 0.50, the normalized clustering coefficient (γ) was >1, the normalized characteristic path length (λ) was ≈1, and the small world properties (σ) was >1 in the TLE group and NC group, which indicates that the structural covariance network of the two groups conforms to the characteristics of “small world” (Figure 1).

**Figure 2:**
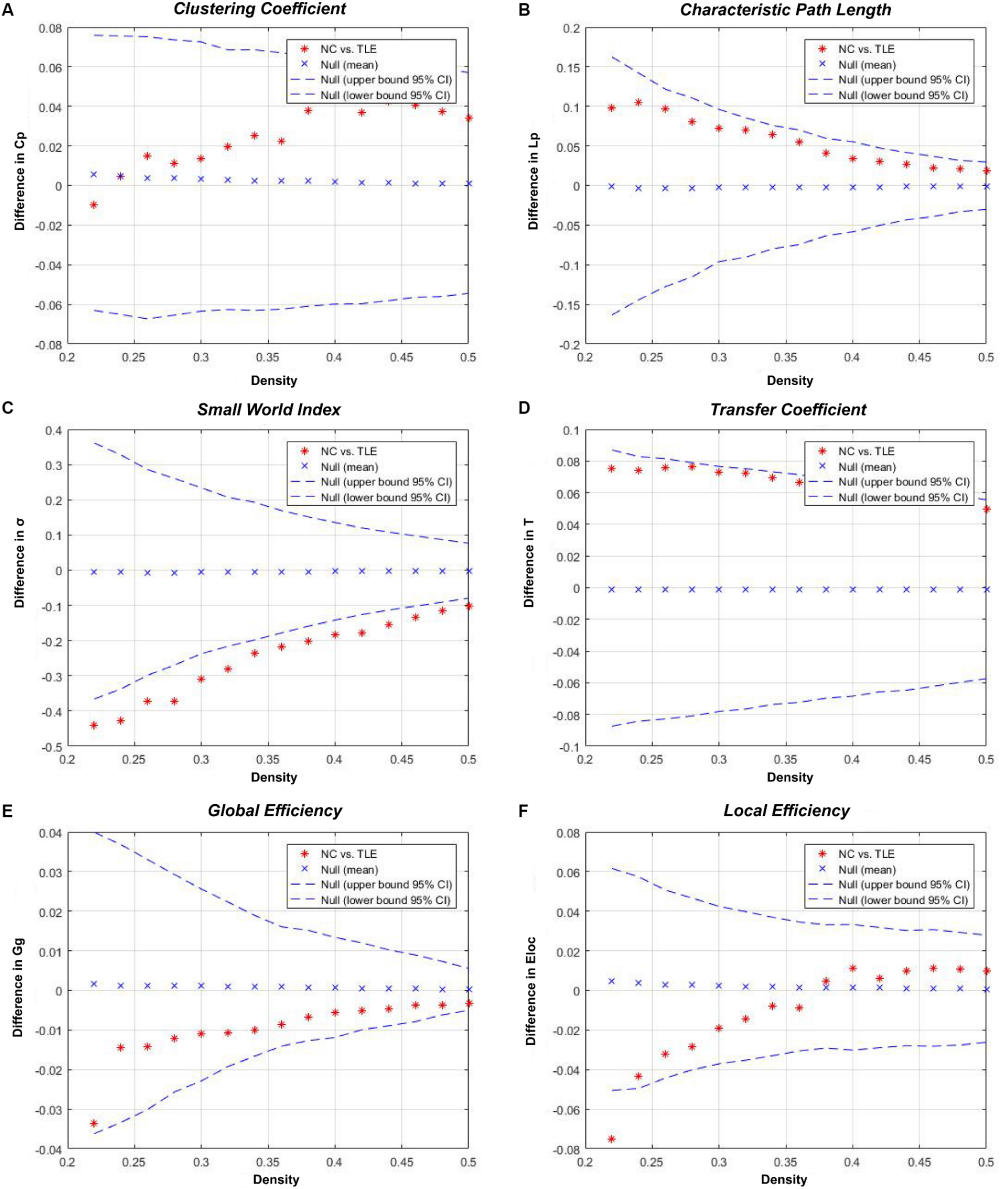
Inter-group differences in the global network metrics of the TLE and NC group in the 0.22–0.50 network density range. The 95% confidence interval and inter-group difference of the **(A)** clustering coefficient, **(B)** shortest path length, **(C)** small world index, **(D)** transfer coefficient, **(E)** global efficiency, and **(F)** local efficiency are shown. The red stars indicate differences between the TLE and NC groups; red stars outside the confidence interval indicate significant inter-group differences at the network density (*p* < 0.05). Positive values indicate TLE group > NC group, negative values indicate TLE group < NC group.

When compared to the NC group, TLE group showed a significant increase in σ (*p* < 0.001) and γ (*p* < 0.001, Cohen’s *d* = 3.205), and a significant decrease in T. These differences between the two groups were statistically significant (*p* < 0.001, Cohen’s *d* = 1.750). Aside from these three indicators, while differences were present between groups, they did not reach statistical significance (*p* > 0.05).Details were shown in Table 2 and Figure 2.

**Table 2:** Comparison between groups of Graph Theory Index parameters under different densities.

Parameters	NC (*n* = 15)	TLE (*n* = 15)	Statistic	*p*	Cohen’s *d*
Eg, Mean ± SD	0.66 ± 0.06	0.67 ± 0.06	*t* = −0.46	0.649	0.167
Eloc, Mean ± SD	0.80 ± 0.04	0.82 ± 0.02	*t* = −0.88	0.391	0.625
Lp, Mean ± SD	1.75 ± 0.18	1.69 ± 0.15	*t* = 0.91	0.371	0.361
γ, Mean ± SD	1.03 ± 0.01	1.28 ± 0.11	*t* = −8.60	<0.001^***^	3.205
T, Mean ± SD	0.66 ± 0.04	0.59 ± 0.04	*t* = 4.55	<0.001^***^	1.750
σ, M (Q₁, Q₃)	1.08 (1.06, 1.10)	1.29 (1.21, 1.43)	*Z* = −5.57	<0.001^***^	
λ, M (Q₁, Q₃)	1.04 (1.03, 1.07)	1.03 (1.01, 1.05)	*Z* = −1.49	0.137	

*t*, *t*-test; Z, Mann–Whitney test; SD, standard deviation; M, Median; Q₁, 1st quartile; Q₃, 3st quartile; Eg, global efficiency; Eloc, local efficiency; γ, normalized clustering coefficient; Lp, characteristic path length; T, transfer coefficient; σ, small world properties; λ, normalized characteristic path length; ^***^p < 0.001.

### Comparison of regional network metrics

**Figure 3:**
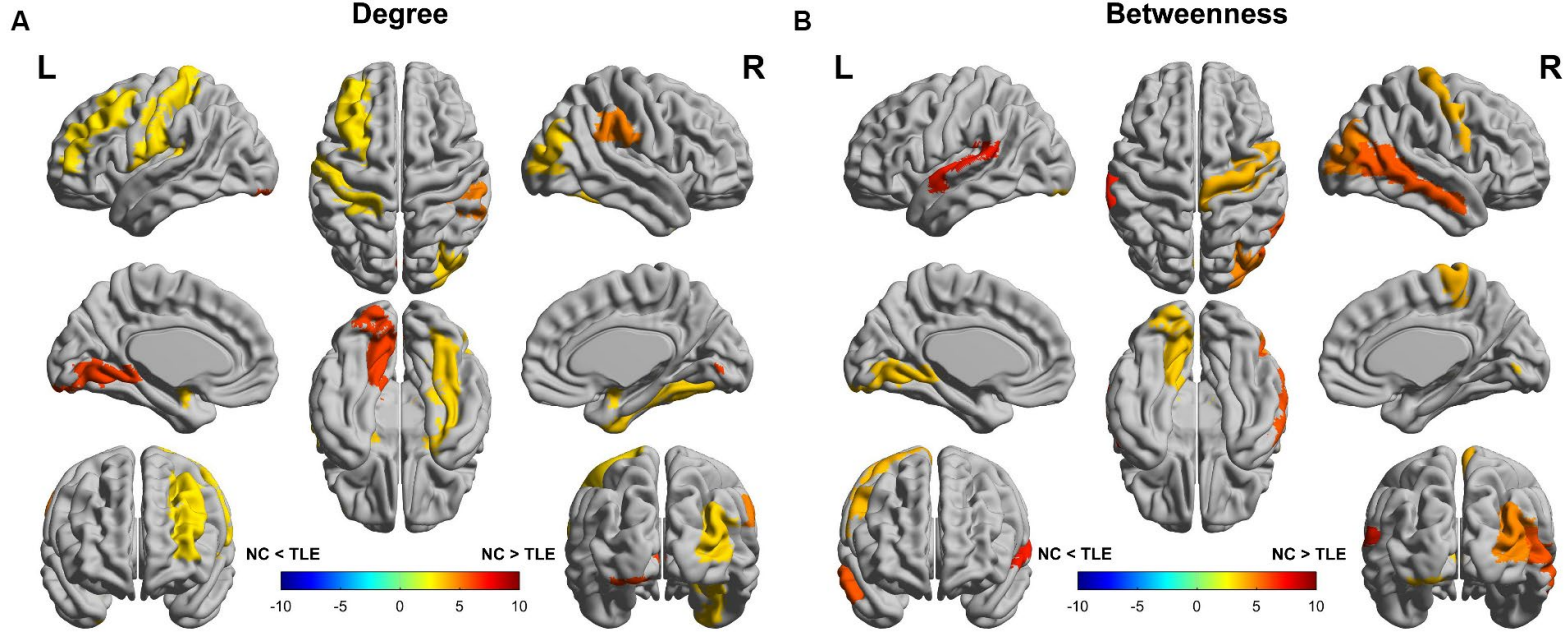
Differences between the TLE and NC groups in regional network metrics (uncorrected). Regions that showed significant differences between TLE and NC in nodal degree **(A)** and nodal betweenness centrality **(B)** for networks thresholded at the minimum density. Warm colors denote regions with significantly higher nodal degree or betweenness in the TLE group compared with the NC group, while cool colors denote regions with significantly higher nodal degree or betweenness in the NC group compared to the TLE group.

We compared nodal degree and nodal betweenness at the minimum network density between the TLE and NC groups. The TLE patients showed a decreased nodal degree in Amygdala_L, Amygdala_R, Frontal_Mid_L, Fusiform_R, Lingual_L, Occipital_Mid_R, Postcentral_L, Putamen_R, Rolandic_Oper_L, SupraMarginal_R and Thalamus_R (*p* < 0.05, uncorrected) compared to the NC group (Figure 3A). The TLE group patients showed a decreased nodal betweenness in Lingual_L, Occipital_Mid_R, Paracentral_Lobule_R, Precentral_R, Temporal_Mid_R, Temporal_Sup_L and Thalamus_R (*p* < 0.05, uncorrected) compared to the NC group (Figure 3B). Although there were no significant differences between the two groups in terms of nodal degree and nodal betweenness even after multiple comparison corrections (FDR, *p* > 0.05), TLE group patients showed both a downward trend in nodal betweenness and noda degree in Occipital_Inf_R, SupraMarginal_R and Temporal_Mid_L compared with the NC group (*p* < 0.05, uncorrected). For details of brain regions, please refer to Supplementary Table S3.

### Degree distribution

**Figure 4:**
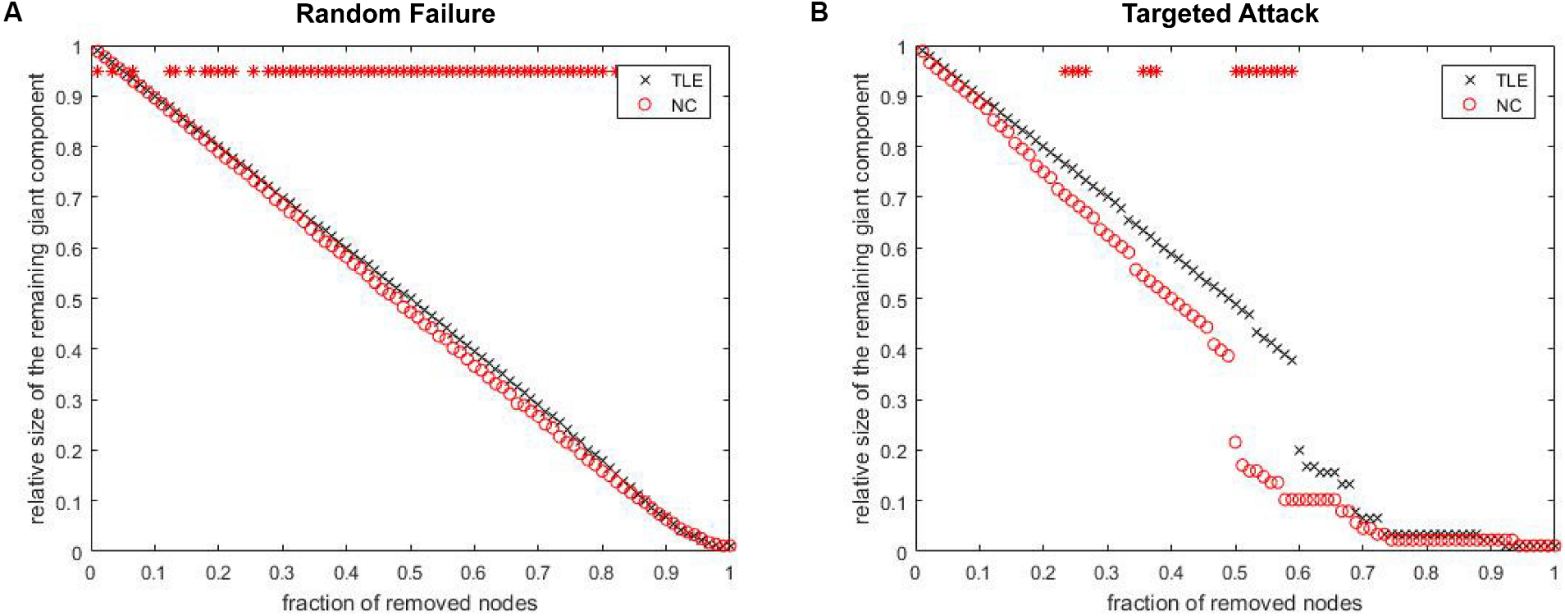
Network Degree distributions. The log–log plot of cumulative degree distributions of **(A)** TLE and **(B)** NC networks at threshold of 0.22 density. The rad line indicates the exponentially truncated power-law curve which was fitted to the cumulative degree distribution of the networks (blue line) Brain Imaging and Behavior.

As shown in Figure 4, the connectivity degree distribution in both the TLE and NC groups met an exponentially truncated power law (Park et al., 2017). For the TLE, the exponent estimate (e) was 1.23 and for NC is 1.04. The cutoff degree (dc) was 7.91 for TLE and 20.49 for NC. The R2 value is 0.97 in both the TLE and NC groups. The R2 was 0.98 for TLE and 0.97 for NC.

### Difference in network resistance between the TLE and NC group

**Figure 5:**
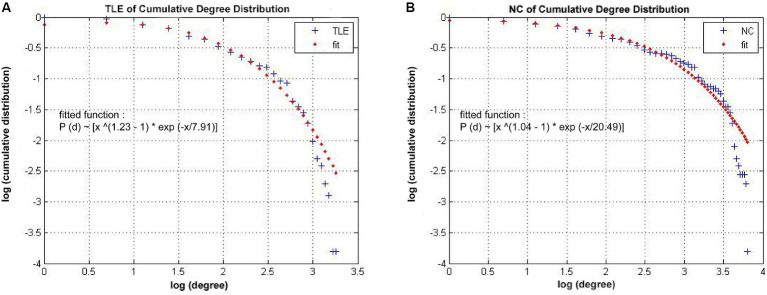
The maximum remaining component size in the network after random failures and targeted attacks. The red point indicate that the difference in the remaining maximum component of the network, between groups at which the node is deleted, is statistically significant.

The analysis of network fault tolerance and anti-aggression revealed that the structural covariance network in TLE patients had significantly higher resistance to random faults compared to the NC group. However, the TLE group had significantly higher resistance to targeted attacks than the NC group for 22–28%, 34–38% and 50–60% of the deleted nodes (Figure 5). The AUC analysis in GAT confirmed that there was a statistically significant difference between the two groups in terms of resistance to random failures (*p* = 0.01) and targeted attacks (*p* = 0.03).

## Discussion

We constructed and compared the properties of the brain GM structural networks between patients with TLE and NC. In our study, we discovered that despite the structural covariance network of TLE patients adhering to features of a small-world network, the topological characteristics of the structural covariance network seem anomalous, evident in a significant increase in sigma (σ) and heightened susceptibility to random failures. Compared to the NC group, not only were global network parameters altered in TLE patients, but the regional network parameters were also affected, marked by a decrease in nodal degree and betweenness. These results suggest that the topological properties of the structural covariance network in patients with TLE are reorganized, which provides new evidence for the neurobiological mechanism of cognitive impairment in patients with TLE from the perspective of the connectome.

### TLE-related alterations in global network parameters

In this study, we found that the structural covariance network of moderate-to-severe TLE patients conforms to small-world characteristics. A small-world network incorporates characteristics of a regular network, denoted by high clustering, along with a random network’s typical short path lengths. This unique blend potentially allows for global interconnectivity while preserving substantial local connections. Our study’s findings indicate that this small-world topology is an integral constituent of the structural organization of brain networks.

Although small-world features exist in adult TLE structural covariance networks, there are significant intergroup differences in terms of multiple global network parameters. Relative to the NC group, the TLE group displayed considerably higher values of γ and σ (*p* < 0.001). These findings indicate a noticeable change in the structure and function of the brain network in patients with temporal lobe epilepsy, characterized by enhanced *γ* and small world propertiess. The standardized clustering coefficient, a measure of interconnectivity between a node’s neighboring nodes (Vecchio et al., 2014; Xu et al., 2019), was elevated. This higher clustering coefficient underscores a robust local connective structure within the network, reflecting advanced local network interactions in TLE patients. An increase in the standardized clustering coefficient would suggest an expanded array of local circuits and clumps within the temporal lobe epilepsy brain network. While these structures facilitate local information processing, they could obstruct global information integration (Fornito et al., 2015), potentially leading to cognitive impairment. However, it was observed in this study that the Eg of the TLE group remained unaltered. This could be attributed to the maintenance of overall network efficiency via compensatory mechanisms or the limited sample size utilized in the study. The Small World Propertie, here is a refined version of the text enclosed in the brackets:

The small world properties, defined as the ratio of the standardized clustering coefficient to the standardized shortest path, laid bare distinct insights during this study. The standardized clustering coefficient (*γ*) of the TLE group was significantly higher than that of the NC group; however, no statistical variance was detected in the standardized shortest path. The small world properties essentially embodies a unique connectivity pattern among the nodes in the network – a pattern that is highly aggregated and, paradoxically, dispersed. The nodes in the network establish connections not merely with neighboring nodes, but also with nodes that are located distantly. This dual connectivity model endows the network with outstanding efficiency in information transmission and robustness (van den Heuvel and Hulshoff Pol, 2010). An elevation in small world propertiess may denote an intensified connection within certain regions of the brain – a compensatory measure taken in response to the disruption of some functions or pathways. This implies that the brain may attempt to counteract cognitive impairments by reorganizing its network structure (Yan et al., 2017; Lin et al., 2020).

In the TLE group, T, was observed to be lower compared to the NC group, with statistical significance noted between the two groups (*p* < 0.001). The transfer coefficient is an indicator of the information relay efficiency between different regions within the brain network (Vecchio et al., 2014; Xu et al., 2019). A decreased transfer coefficient in the TLE group could potentially be attributed to seizure-induced disruptions in network connectivity. Seizures can instigate an excessive neuronal discharge (Engel, 1983), thereby impairing neuronal connections and weakening overall information relay efficiency within the brain network. Prior research has affirmed that slow processing, a vital cognitive side effect of TLE, is closely linked with alterations in brain structure and connectivity (Hwang et al., 2019). Hence, this study proposes that the observed decrease in the transfer coefficient underpins the detrimental impacts of seizures on brain network connectivity in patients with TLE.

Therefore, the change in global topological properties associated with TLE may reflect a less ideal topological organization, which provides insight for understanding the relationship between network topological properties and the neuropathological state of the disease.

### TLE-related alterations in regional network parameters

We found abnormal regional network parameters in multiple regions of the brain showing both decreased nodal betweenness/degree in TLE patients compared with the NC group. Although these results were not statistically different after a correction for multiple comparisons, we should note that TLE patients showed both a decreased nodal betweenness and degree in the Lingual_L, Occipital_Mid_R and Thalamus_R compared with the NC group, indicating that there is a downward trend in nodal betweenness and degree in these brain regions.

The Lingual Gyrus, nestled in the medial temporal lobe, forms an integral part of the cerebral cortex (Powell et al., 2004; Sundram et al., 2010; Sormaz et al., 2017; Zanao et al., 2021). Positioned adjacent to the hippocampus, it intimately involves in processes like memory formation, storage, retrieval, regulation of emotions, and sensory processing (Powell et al., 2004; Sundram et al., 2010; Sormaz et al., 2017; Zanao et al., 2021). The Lingual Gyrus also contributes to the operation of the Default Mode Network (DMN), a network exhibiting activity during restful states and is implicated in functions such as introspection and mental operations (Zanao et al., 2021). This study unveiled a decline in both the nodal betweenness and degree of the left lingual gyrus in the TLE group, consistent with the abnormalities in the Lingual Gyrus proposed by fMRI graph theory analysis (Trimmel et al., 2021). The downslide in the nodal betweenness and degree within the Lingual Gyrus points to an alteration in brain network connectivity amongst TLE patients. This anomaly may indicate the potential causes of negative emotions (as per depression scales) and cognitive impairments (indicated by low MoCA scores), however, we were unable to further analyze the relationship between the abnormalities in local network parameters and the degree of these pathological changes.

The Middle Occipital Gyrus, strategically situated on the medial surface of the brain’s occipital lobe, adopts a predominantly horizontal orientation, occupying the space between the Superior and the Inferior Occipital Gyri (Renier et al., 2010). Acting as the domicile for the primary visual cortex, the Middle Occipital Gyrus marks the onset of visual information processing. Its primary function includes assimilating visual signals from the retinas of both eyes, thereby initiating their processing. The Middle Occipital Gyrus exhibits a pivotal association with visual perception, attention, and cognition (Renier et al., 2010; Xiong et al., 2016; Hwang et al., 2019). Through an analysis based on VBM, Kim et al. (2016) proposed a noteworthy reduction in the volume of the Middle Occipital Gyrus in patients afflicted with right temporal lobe epilepsy. This contraction in volume potentially disrupts its transmission and processing capacities within the network, thereby instigating a varying degree of decrement in the nodal betweenness and degree of the Middle Occipital Gyrus. The observations from this study lend credibility to this surmise. An examination of the brain structure image using graph theory instigates the revelation of abnormalities in the network parameters of the Middle Occipital Gyrus. This could potentially unmask the underlying causes of cognitive anomalies (as measured by MoCA and MMSE) and anxiety (as evaluated by HAMA) prevalent within the TLE group from a network topology perspective.

The Thalamus, a crucial structure centrally located within the brain, is paramount to the transmission of information. Comprising various distinct nuclei, it is instrumental in both the transmition and integration of neural signals, thereby influencing sensory and motor functions as well as levels of consciousness (Sieb, 1990; Romero-Romo et al., 2010). As the primary relay station for sensory information (excluding smell), the Thalamus is tasked with forwarding these signals to the appropriate areas of the cerebral cortex (Wilf et al., 2016). Moreover, the Thalamus forms a complex network functionality with other brain regions, including the cortex, basal ganglia, and brain stem. This interplay is vital for the regulation of attention, emotion, and memory (Hirata and Castro-Alamancos, 2010; Ramkiran et al., 2019). The Thalamus’ significance in the pathogenesis of temporal lobe epilepsy has been well-established (Labate et al., 2008), numerous VBM studies have indicated variances in the shrinkage of the Thalamus in patients with temporal lobe epilepsy (Keller and Roberts, 2008; Labate et al., 2008; Riederer et al., 2008; Jber et al., 2021). Being rich in neurons, any reduction in the volume of the grey matter could likely trigger a direct descent in nodal betweenness and degree in the middle temporal gyrus. This study observed a decrement in the nodal betweenness and degree of the left middle temporal gyrus in TLE, which aligns with the aforementioned premise. Being a functional execution and operation center, the decline in thalamic local network parameters suggested by graph theory analysis may unveil the complications facing patients with TLE, such as cognitive impairment and depression.

### Degree distribution

Our study revealed that both individuals with TLE and normal controls (NC) exhibited grey matter networks that followed an exponentially truncated power law distribution (Lambiotte et al., 2012). This means that the structural networks consist of numerous nodes with relatively low degree and a few nodes with high degree (hub nodes). Although the exact biological cause of this network topology remains unclear, it is possible to describe mechanisms of neuronal development using the concept of exponentially truncated power-law mechanisms, which reflect the physical limitations on afferent connections that neurons can support (Sporns et al., 2004; Lambiotte et al., 2012). Additionally, the slightly altered cutoff degree of the distribution in the TLE network, compared to NC, may indicate a rearrangement of afferent connections.

### TLE-related alterations in network resistance

Our results showed the stronger stability of random faults in the structural covariance network of TLE patients. Previous studies had shown that small-world networks with core nodes are highly resistant to random failures and targeted attacks (Stam et al., 2006; He et al., 2008). In a previous analysis of structural graph theory in other diseases, it was found that the high vulnerability of random faults in the structural covariance network of patients (Cao et al., 2019; Liu et al., 2020). However, the results of this study contradicted this finding, which may reflect the uniqueness of epilepsy. The TLE patients’ SCN demonstrated significantly higher resistance to random failures compared to the NC group, which may be attributed to two possible reasons: First, the neural networks in TLE patients might possess more redundancy or compensatory mechanisms (Chang et al., 2012; Jiang et al., 2021), which help maintain function and reduce failures. Second, these networks could have adapted to epileptic activity (neuronal abnormal discharges) (Engel, 1983), thereby conferring greater resilience to random perturbations. Furthermore, our study also observed that the fault tolerance of SCNs in TLE tends towards more regular networks, aligning with previous findings suggesting that regular networks may exhibit weaker resistance to pathological assaults (Friston et al., 2007). From a connectomics perspective, lower network fault tolerance may also provide neuroimaging evidence for the increased risk of neural damage and cognitive impairments in patients with TLE. In comparison to NC, TLE patients demonstrated significantly reduced scores in MoCA, MMSE, HAMA, and HAMD. These findings indicate that TLE may be more susceptible to pathological conditions such as anxiety, depression, and cognitive impairments, potentially due to the diminished fault tolerance of TLE’s structural covariance networks. However, this study was not without limitations; we were unable to further analyze the specific correlation between the network fault tolerance of individual TLE patients and these clinical scale scores. Consequently, this study merely proposed a hypothesis rather than drawing a rigorous conclusion.

### Limitations

While this study reports some positive findings, there are several limitations that need to be acknowledged. Firstly, the research was conducted with a small sample size and the local network parameters were not corrected. As a result, future studies necessitate larger sample sizes to validate our outcomes. Secondly, this study was unable to obtain the relevant network parameters of individual patients; therefore, it could not delve into the relationship between clinical scale scores and network changes. In future research, we will attempt other methods to obtain brain network parameters at the individual level, hoping to further understand the changes in the neurobiological mechanisms of TLE patients. Lastly, our study was confined to constructing a group-level structural covariance network and did not take into account an individual-level network. This omission restricts the potential to further investigate the associations between network parameters and clinical variables.

## Conclusion

To conclude, this study demonstrated that structural covariance networks in TLE patients were abnormal according to multiple network parameters. These findings revealed abnormalities in temporal lobe epilepsy from the perspective of network connectivity, which may promote our understanding of the neurobiological mechanisms in patients with temporal lobe epilepsy.

## Data availability statement

The original contributions presented in the study are included in the article/Supplementary material, further inquiries can be directed to the corresponding author.

## Author contributions

YC: Writing – original draft, Writing – review & editing, Conceptualization, Data curation, Formal analysis, Investigation, Methodology, Project administration, Resources, Software, Supervision, Validation, Visualization. LS: Conceptualization, Data curation, Formal analysis, Investigation, Methodology, Project administration, Resources, Software, Supervision, Validation, Visualization, Writing – original draft, Writing – review & editing. SW: Conceptualization, Data curation, Formal analysis, Investigation, Methodology, Project administration, Resources, Software, Supervision, Validation, Visualization, Writing – original draft, Writing – review & editing. BG: Conceptualization, Data curation, Formal analysis, Investigation, Methodology, Project administration, Resources, Software, Supervision, Validation, Visualization, Writing – original draft, Writing – review & editing. JP: Conceptualization, Data curation, Formal analysis, Investigation, Methodology, Project administration, Resources, Software, Supervision, Validation, Visualization, Writing – original draft, Writing – review & editing. YQ: Conceptualization, Data curation, Formal analysis, Investigation, Methodology, Project administration, Resources, Software, Supervision, Validation, Visualization, Writing – original draft, Writing – review & editing. YL: Conceptualization, Data curation, Formal analysis, Investigation, Methodology, Project administration, Resources, Software, Supervision, Validation, Visualization, Writing – original draft, Writing – review & editing. NY: Conceptualization, Data curation, Formal analysis, Investigation, Methodology, Project administration, Resources, Software, Supervision, Validation, Visualization, Writing – original draft, Writing – review & editing. HL: Data curation, Formal analysis, Investigation, Methodology, Project administration, Resources, Software, Supervision, Validation, Visualization, Writing – original draft, Writing – review & editing, Conceptualization. YW: Conceptualization, Data curation, Formal analysis, Investigation, Methodology, Project administration, Resources, Software, Supervision, Validation, Visualization, Writing – original draft, Writing – review & editing. BS: Conceptualization, Data curation, Formal analysis, Investigation, Methodology, Project administration, Resources, Software, Supervision, Validation, Visualization, Writing – original draft, Writing – review & editing.

## Footnotes

^1^ http://dbm.neuro.uni-jena.de/vbm

^2^ https://www.r-project.org/
