# Supplementary material for: Topological regularization of networks in temporal lobe epilepsy: a structural MRI study
Source: Front Neurosci. 2024 Jul 4;18:1423389. doi: 10.3389/fnins.2024.1423389 (PMC11259028; doi:10.3389/fnins.2024.1423389)
Supplement: Supplementary file 1 [file Data_Sheet_1.docx]

Supplementary Material

**Topological Regularization of Networks in temporal lobe epilepsy: A Structural MRI Study**

**Supplementary Methods**

**Network analyses**

***Small world parameters***

The small-worldness of a complex network has two crucial metrics: the clustering coefficient (*C_p_*) and the characteristic path length (*L_p_*). *C_p_* is taken as a measure of functional segregation and *L_p_* is taken as a measure of functional integration (1).

1. The clustering coefficient *C_i_* of a node i is defined as the number of existing links divided by the number of all possible links among the neighbors of a node:

$$C_{i}=\frac{2E_{i}}{K_{i}(K_{i}-1)}$$

where *K_i_* is the number of connections to node i, *E_i_* is the number of existing connections among the neighbors. The clustering coefficient of a network is the average of the clustering coefficient of all nodes:

$$C_{p}=\frac{1}{N}\sum_{i\in G} G_{i}$$

which is a measure of the extent of local cliquishness or local efficiency of information transfer of a network (2).

1. The shortest path length of a node in the network G (N, E) is defined as:

$$L_{i}=\frac{1}{N-1}\sum_{i\neq j\in G} d_{ij}$$

in which *d_ij_* is the shortest absolute path length between the i and j nodes. *L_p_* is the average of the shortest path length between the nodes:

$$L_{p}=\frac{1}{N}\sum_{i\in G} L_{i}$$

which quantifies the extent of average connectivity or the overall routing efficiency of the network (3).

***Network measures***

*Global measures*

Global efficiency (*E_glob_*) and mean local efficiency (*E_loc_*) were adopted to characterize the global topological organization of brain networks. Efficiency is a biologically relevant metric to describe brain networks from the perspective of parallel information flow that can deal with either the disconnected or nonsparse graphs or both (2).

1. The global efficiency *E_glob_* is defined as follows:

$$E_{glob}=\frac{1}{N(N-1)}\sum_{j\neq i\in G} \frac{1}{L_{ij}}$$

where *L_ij_* is the shortest path length between nodes i and j. *E_glob_* serves as a measure of parallel information transmission in the entire network (3).

1. The local efficiency of *G* is measured as follows:

$$E_{loc}=\frac{1}{N}\sum_{i\in G} E_{glob}(i)$$

where *E_glob_*(i) is the global efficiency of *G_i_*. *G_i_* is a subgraph that includes the nodes that connect to node i. Local efficiency measures the fault tolerance of the network, indicating the capacity for information exchange within each subgraph when the index node is eliminated (3).

*Regional nodal measures*

To examine the nodal characteristics of structural brain network, we considered four nodal metrics: nodal degree (*K_i_*), nodal betweenness (*B_i_*), nodal clustering coefficient (*C_i_*) and nodal local efficiency (*E_i_*)

1. *K_i_* represents the number of connections to a node, which is defined as follows (4):

$$K_{i}=\sum_{i\neq j\in G} e_{ij}$$

where *e_ij_* is the (i,j)th element in the binary correlation matrix.

1. *B_i_* is defined as follows(5):

$$B_{i}=\sum_{i\neq j\neq k\in G} \frac{\delta_{jk}(i)}{\delta_{jk}}$$

Where *δ_jk_* is the number of shortest paths from node j to node k and *δ_jk_*(i) is the number of shortest paths from node j to node k that pass through node i within graph *G*. *B_i_* measures the quantity of information transmitted via node i between the rest node and the entire network.

1. *C_i_* is defined as follows (6):

$$C_{i}=\frac{2E_{i}}{K_{i}(K_{i}-1)}$$

where *K_i_* is the number of connections to node i, *E_i_* is the number of existing connections among the neighbors.

1. *E_i_* is defined as follows (3):

$$E_{i}=\frac{1}{N-1}\sum_{i\neq j\in G} \frac{1}{L_{ij}}$$

where *L_ij_* is the shortest path length between nodes i and j.

**Table S1. Detailed Information on Different Device Groups**

|  |  | uMR Omega 3.0T(n = 43) |  | Philips Ingenia CX 3.0T(n = 39) |
| --- | --- | --- | --- | --- |
|  | TLE (n = 17) | NC (n = 26) | TLE (n = 18) | NC (n = 21) |
| Gender, n(%) |  |  |  |  |
| Male | 8 (47.06%) | 12 (46.15%) | 11 (61.11%) | 7 (33.33%) |
| Female | 9 (52.94%) | 14 (53.85%) | 7 (38.89%) | 14 (66.67%) |
| Age | 47.65 ± 12.79 | 64.35 ± 5.31 | 44.00 (34.25, 51.00) | 30.00 (25.00, 37.00) |

**Table S2. Definition and meaning of global attribute parameters of brain network**

| Parameters | Definition | Meaning |
| --- | --- | --- |
| Cp | Possibility of neighbors of network nodes being neighbors to each other | To measure the network collectivization, the network with high clustering coefficient means that the local nodes of the network are more closely connected |
| Lp | The average of the shortest paths for a network node to reach other nodes in the network | The shorter the shortest path, the shorter the path that nodes need to pass to transmit information, thus saving system resources more |
| γ | The ratio of clustering coefficient of real network to clustering coefficient of 100 random networks | To gauge the network's 'separation' function |
| λ | The ratio of the shortest path of a real network to the shortest path of 100 random networks | To determine the 'integration' function of the network |
| σ | The ratio of normalized clustering coefficient to standardized shortest path | To consider the balance between both 'separation' and 'integration' functions within the network |
| T | An indicator of the information relay efficiency between different regions within the brain network | To signify communication and interaction amongst various brain regions |
| Eg | Average global efficiency of all nodes | To measure the global information transmission capacity of the network, the higher the global efficiency of the network, the faster the information transmission rate within the whole network |
| Eloc | Average local efficiency of all nodes | To measure the local information transmission capacity of the network, the higher the local efficiency of the network, the faster the information transmission rate within the whole network |

Cp: clustering coefficient; Lp: characteristic path length; γ: normalized clustering coefficient; λ: normalized characteristic path length; σ: small world properties; T: transfer coefficient; Eg: global efficiency; Eloc: local efficiency.

**Table S3. Abbreviations for node**

| **Abbs** | **Regions** | **Abbs** | **Regions** |
| --- | --- | --- | --- |
| Amygdala_L | Left Amygdala | SupraMarginal_R | Right SupraMarginal |
| Amygdala_R | Right Amygdala | Thalamus_R | Right Thalamus |
| Frontal_Mid_L | Left Middle Frontal | Paracentral_Lobule_R | Right Paracentral Lobule |
| Fusiform_R | Right Fusiform | Precentral_R | Right Precentral |
| Lingual_L | Left Lingual | Temporal_Mid_R | Right middle Temporal |
| Occipital_Mid_R | Right Middle Occipital | Temporal_Sup_L | Left Superior Temporal |
| Postcentral_L | Left Postcentral | Occipital_Inf_R | Right Occipital Inferior |
| Putamen_R | Right Putamen | Temporal_Mid_L | Left middle Temporal |
| Rolandic_Oper_L | Left Rolandic Operculus |  |  |

**Reference：**

1. Watts DJ, Strogatz SH. Collective dynamics of 'small-world' networks. *Nature* (1998) 393: 440-442. doi:Doi 10.1038/30918

2. Latora V, Marchiori M. Efficient behavior of small-world networks. *Physical Review Letters* (2001) 87. doi:ARTN 19870110.1103/PhysRevLett.87.198701

3. Achard S, Bullmore E. Efficiency and cost of economical brain functional networks. *PLoS Comput Biol* (2007) 3: e17. doi:10.1371/journal.pcbi.0030017

4. Sang L, Zhang J, Wang L, Zhang J, Zhang Y, Li P, et al. Alteration of Brain Functional Networks in Early-Stage Parkinson's Disease: A Resting-State fMRI Study. *PLoS One* (2015) 10: e0141815. doi:10.1371/journal.pone.0141815

5. Freeman LC. Set of Measures of Centrality Based on Betweenness. *Sociometry* (1977) 40: 35-41. doi:Doi 10.2307/3033543

6. Xu J, Zhang J, Zhang J, Wang Y, Zhang Y, Wang J, et al. Abnormalities in Structural Covariance of Cortical Gyrification in Parkinson's Disease. *Front Neuroanat* (2017) 11: 12. doi:10.3389/fnana.2017.00012
